# Growth, nutritional status, and physical fitness trajectories among primary and secondary school students and their associations with poor vision: a school-based study in Chongqing, China

**DOI:** 10.3389/fpubh.2026.1948162

**Published:** 2026-10-13

**Authors:** Yaocheng Luo, Wenhua Ran, Shaocheng Fan, Yuanyuan Lan, Youtao Mou, Ling Liu, Yong Zhao

**Affiliations:** 1 School of Public Health, Chongqing Medical University, Chongqing, China; 2 Research Center for Medicine and Social Development, Chongqing Medical University, Chongqing, China; 3 Research Center for Public Health Security, Chongqing Medical University, Chongqing, China; 4 Nutrition Innovation Platform-Sichuan and Chongqing, School of Public Health, Chongqing Medical University, Chongqing, China; 5 Yuzhong District Primary and Secondary School Health Care Center, Chongqing, China

**Keywords:** body mass index, physical examination, poor vision, primary and secondary school students, vital capacity index

## Abstract

**Purpose:**

To examine trends in growth and development, physical fitness, and visual acuity indicators among primary and secondary school students in Yuzhong District, Chongqing, and to explore the associations of body mass index (BMI) and vital capacity index (VCI) with poor vision.

**Methods:**

Physical examination data of primary and secondary school students in Yuzhong District, Chongqing, from 2022 to 2026 were used to describe growth and development, physical fitness, and visual acuity indicators. Group-based trajectory modeling (GBTM) was applied to identify distinct trajectories of BMI and VCI. Logistic regression was used to analyze the associations between BMI and VCI trajectories and the detection rate of poor vision, as well as potential nonlinear relationships.

**Results:**

From 2022 to 2026, height, weight, and VCI increased among primary and secondary school students in Yuzhong District, Chongqing, while obesity prevalence declined (all trend *p* < 0.001). Poor vision increased from 73.3 to 75.0%. GBTM identified three trajectories for BMI and four trajectories for VCI. Compared with the low-level increasing BMI trajectory, medium and high-level increasing trajectories were associated with 13.4 and 36.8% higher odds of poor vision, respectively. For VCI, the medium-level decreasing trajectory was associated with 11.7% higher odds, whereas the high-level decreasing trajectory was associated with a 37.1% lower risk.

**Conclusion:**

From 2022 to 2026, growth and development indicators among primary and secondary school students in Yuzhong District improved, while poor vision remained prevalent. BMI and VCI trajectories were associated with poor vision, highlighting the importance of integrated monitoring and targeted interventions.

## 1 Introduction

The physical and mental health of children and adolescents has received increasing attention. As an important public health system for the systematic monitoring of growth and development and common diseases, student health examinations provide a critical basis for identifying and preventing health problems. Poor vision, driven by intensive visual demands and unhealthy eye-use habits, has become a major global public health issue, with the situation in China being particularly severe. A recent meta-analysis published in 2025 showed that the prevalence of myopia among children and adolescents in China has reached 58%, indicating that the outlook for prevention and control remains far from optimistic (1).

Studies have shown that visual health is closely related to overall growth and development as well as physical function in children and adolescents (2). Physical development and fitness may influence visual development through physiological maturation, metabolism, and behavioral patterns (3–5). Among relevant indicators, body mass index (BMI) and vital capacity index (VCI) are commonly used to assess growth and development, nutritional status, and physical fitness. Previous studies have found that increased BMI is associated with a higher risk of myopia (6), while higher levels of physical activity may reduce the risk of myopia; by contrast, sedentary behavior and prolonged near-work activities increase the risk of myopia (7, 8).

Against the backdrop of increasingly prominent health problems among children and adolescents nationwide, Yuzhong District of Chongqing has attached great importance to student health and related prevention and control efforts. During the 2021–2022 academic year, the detection rate of overnutrition among primary and secondary school students aged 6–18 years in Yuzhong District was 34.71%, while the detection rates of poor vision among male and female students aged 6–18 years were 57.51 and 61.34%, respectively (9). To address these serious prevention and control challenges, Yuzhong District successively issued the Implementation Plan for Healthy China Yuzhong Action (Revised Edition) and the Work Plan for Building Yuzhong District into a National Reform Pilot Zone for the Prevention and Control of Myopia among Children and Adolescents, thereby continuously promoting student health and myopia prevention and control. Accordingly, based on health examination data of primary and secondary school students in Yuzhong District, Chongqing, from the 2022 to 2025 academic years, this study analyzed trends in growth and development, physical fitness, and visual acuity indicators, and explored the associations of BMI and VCI with poor vision, aiming to provide scientific evidence for local student health promotion.

## 2 Materials and methods

### 2.1 Study design and population

This school-based study comprised two complementary analytical components: a repeated cross-sectional analysis and a longitudinal trajectory analysis. Health examination data were obtained from primary and secondary school students in Yuzhong District, Chongqing, China, for four consecutive academic years (2022–2023, 2023–2024, 2024–2025, and 2025–2026), provided by the Yuzhong District Institute of School Health Care in Chongqing. A detailed flow diagram illustrating the data screening, cross-year linkage, inclusion and exclusion procedures, and derivation of the analytical samples is presented in Figure 1.

**Figure 1:**
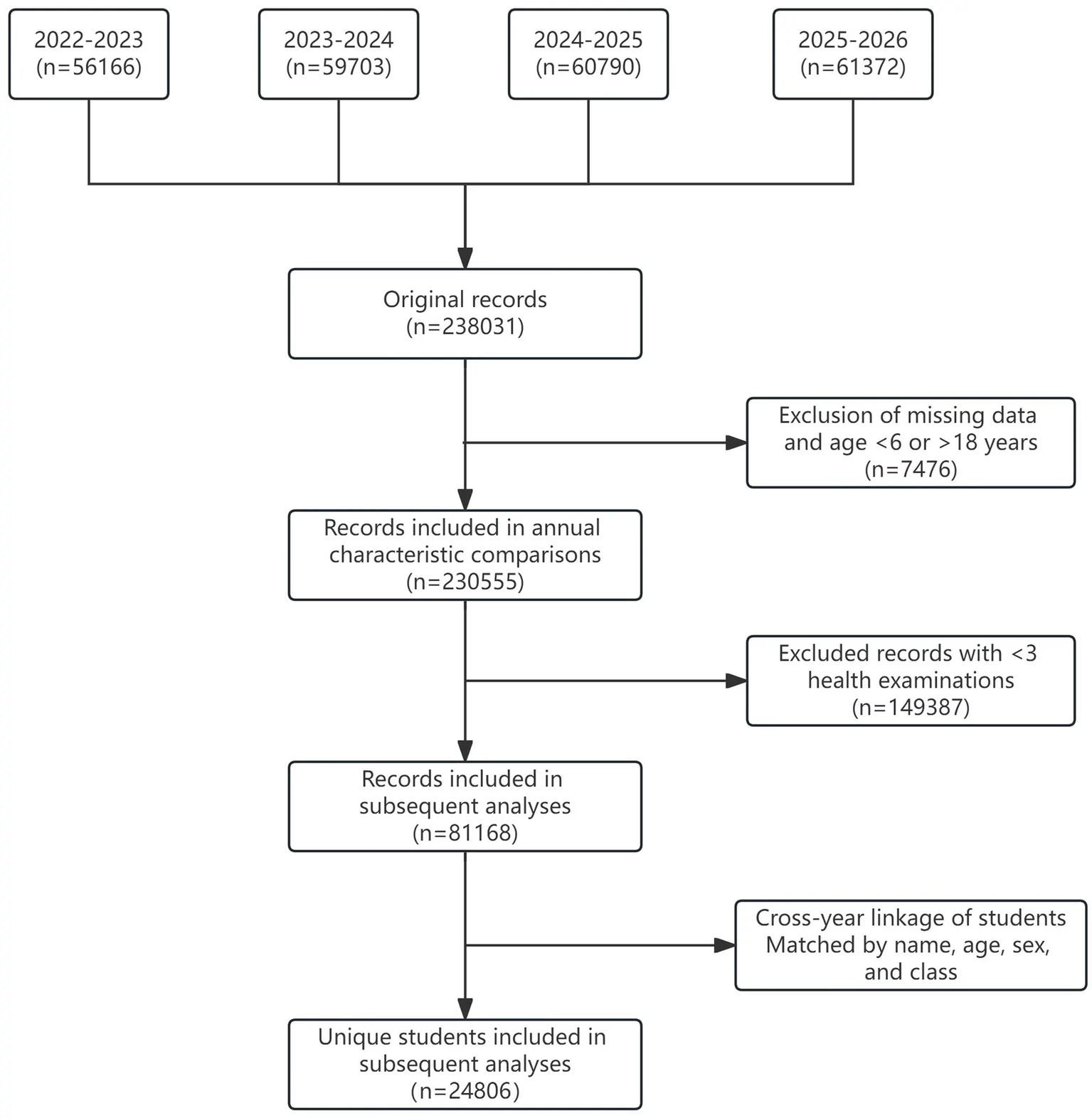
Flowchart of participant selection.

### 2.2 Examination methods

The health examination procedures strictly followed the requirements and specifications of relevant documents, including the Technical Standard for Student Health Examination (GB/T 26343–2010) and the Administrative Measures for Health Examinations of Primary School Students (2021 Edition). Health examinations were conducted regularly each year for primary and secondary schools within the district (10, 11).

### 2.3 Diagnostic criteria

Height and weight were measured, and overweight and obesity status were determined according to the criteria specified in the Screening for Overweight and Obesity among School-Age Children and Adolescents (WS/T 586–2018) (12). Thinness was defined according to the criteria specified in the Screening for Malnutrition among School-age Children and Adolescents (WS/T 456–2014) (13). Vital capacity was measured using an electronic spirometer or a rotary spirometer in accordance with the requirements of the Technical Standard for Student Health Examination (GB/T 26343–2010). Participants were measured in a standing position and performed two chest-expansion movements before measurement. They were then instructed to inhale as deeply as possible and, after reaching full inspiration, exhale forcefully into the mouthpiece at a moderate speed until no further expiration was possible. Each participant underwent two measurements, and the maximum value was recorded as the vital capacity, in milliliters (mL). The vital capacity index (VCI) was calculated as vital capacity (mL) divided by body weight (kg) and expressed in mL/kg. Visual acuity was assessed using a standard logarithmic visual acuity chart for both eyes. Poor vision was defined as an uncorrected distance visual acuity of <5.0 in either eye.

### 2.4 Quality control

Measurement instruments of the same model were used and calibrated before examination. Field procedures strictly followed the Technical Standard for Student Health Examination (GB/T 26343–2010), and all examination items were performed by specialist physicians according to unified standards. The reading error for height measurement did not exceed ±0.5 cm, and that for weight measurement did not exceed ±0.1 kg. Vital capacity was measured twice for each participant.

### 2.5 Statistical analysis

Statistical analyses were performed using R software, version 4.5.1. Continuous variables were described using means and standard deviations, while categorical variables were described using frequencies and percentages. Analysis of variance or chi-square tests were used for between-group comparisons. Linear regression was used to assess trends in continuous variables, while logistic regression was used to assess trends in binary variables. Group-based trajectory modeling (GBTM) was used to identify longitudinal patterns of BMI and VCI across the four academic-year waves (2022–2023, 2023–2024, 2024–2025, and 2025–2026). Students with at least three health examination records across these four waves were included in the trajectory analysis. Models with 1–5 trajectory groups were fitted using second-order polynomial terms with the “gbmt” package (version 0.1.4) in R. Models were compared using AIC, BIC, CAIC, SSBIC, HQIC, average posterior probability of assignment (AvePP), and odds of correct classification (OCC), together with trajectory group size and interpretability. Based on these criteria, three groups were selected for BMI and four groups for VCI. The identified trajectories were treated as categorical independent variables, and logistic regression was used to analyze their associations with poor vision. For the logistic regression analyses, poor vision status assessed at the final examination in the 2025–2026 academic year was used as the outcome. To explore possible nonlinear relationships between BMI, VCI, and poor vision, restricted cubic spline (RCS) analyses were conducted using the same sample (*n* = 24,806) as that included in the GBTM analysis. The mean BMI and VCI across available examination time points for each student were calculated and modeled separately as continuous variables.

## 3 Results

### 3.1 Annual changes in growth and development indicators

Table 1 presents the health examination results of primary and secondary school students in Yuzhong District from 2022–2023 to 2025–2026. Height and weight showed significant overall increasing trends in both boys and girls, although they fluctuated slightly across years. Vital capacity and BMI also fluctuated over the study period but showed significant overall trends (all P for trend < 0.001). The prevalence of thinness increased from 4.4% in 2022–2023 to 9.2% in 2025–2026, whereas the prevalence of overweight decreased from 32.7 to 26.9% and obesity decreased from 15.4 to 11.7% (all P for trend < 0.001). The detection rate of poor vision fluctuated across years but showed an overall increasing trend, rising from 73.3% in 2022–2023 to 75.0% in 2025–2026 (P for trend < 0.001). The population distribution across academic years is presented in Supplementary Table S1.

**Table 1:** Basic characteristics of physical examination indicators for primary and secondary school students in Yuzhong District, 2022–2026.

Indicator	Category	2022–2023	2023–2024	2024–2025	2025–2026	*p* value	*P* for trend
Height	Overall	148.94 (16.8)	149.93 (16.1)	152.00 (16.6)	152.97 (16.5)	<0.001	<0.001
	Boys	150.70 (18.4)	151.66 (17.5)	153.79 (18.2)	154.91 (18.0)	<0.001	<0.001
	Girls	147.03 (14.7)	148.03 (14.1)	150.00 (14.5)	150.78 (14.3)	<0.001	<0.001
Weight	Overall	45.06 (17.5)	44.35 (17.6)	45.73 (17.6)	46.41 (17.8)	<0.001	<0.001
	Boys	47.43 (19.5)	46.62 (19.5)	48.12 (19.6)	48.96 (19.8)	<0.001	<0.001
	Girls	42.48 (14.6)	41.87 (14.7)	43.06 (14.7)	43.52 (14.8)	<0.001	<0.001
VC	Overall	2320.53 (1006.9)	2395.02 (1229.6)	2279.55 (960.1)	2310.14 (976.3)	<0.001	<0.001
	Boys	2554.83 (1170.7)	2627.35 (1401.4)	2500.16 (1100.4)	2534.49 (1109.9)	<0.001	<0.001
	Girls	2065.75 (708.1)	2139.76 (943.8)	2032.84 (695.0)	2056.67 (719.2)	<0.001	<0.001
BMI	Overall	19.58 (6.2)	18.94 (5.0)	19.03 (4.2)	19.07 (4.3)	<0.001	<0.001
	Boys	20.01 (6.3)	19.34 (4.6)	19.46 (4.5)	19.52 (4.5)	<0.001	<0.001
	Girls	19.10 (6.0)	18.50 (5.4)	18.55 (3.8)	18.56 (3.9)	<0.001	<0.001
Thinness	Overall	2,335 (4.4)	5,044 (9.0)	4,846 (8.0)	5,631 (9.2)	<0.001	<0.001
	Boys	1,361 (4.9)	2,932 (10.0)	2,838 (8.9)	3,246 (10.0)	<0.001	<0.001
	Girls	974 (3.8)	2,112 (7.9)	2008 (7.1)	2,385 (8.3)	<0.001	<0.001
Overweight	Overall	17,330 (32.7)	14,891 (26.7)	16,163 (26.8)	16,494 (26.9)	<0.001	<0.001
	Boys	10,790 (39.1)	9,462 (32.4)	10,462 (32.9)	10,778 (33.2)	<0.001	<0.001
	Girls	6,540 (25.7)	5,429 (20.4)	5,701 (20.0)	5,716 (19.9)	<0.001	<0.001
Obesity	Overall	8,171 (15.4)	6,645 (11.9)	7,146 (11.8)	7,180 (11.7)	<0.001	<0.001
	Boys	5,321 (19.3)	4,441 (15.2)	4,775 (15.0)	4,858 (14.9)	<0.001	<0.001
	Girls	2,850 (11.2)	2,204 (8.3)	2,371 (8.3)	2,322 (8.1)	<0.001	<0.001
Poor vision	Overall	38,895 (73.3)	40,856 (73.1)	46,575 (77.2)	45,956 (75.0)	<0.001	<0.001
	Boys	19,583 (70.9)	20,689 (70.7)	23,872 (75.0)	23,736 (73.0)	<0.001	<0.001
	Girls	19,312 (76.0)	20,167 (75.8)	22,703 (79.7)	22,220 (77.3)	<0.001	<0.001

### 3.2 Growth curves of growth and development indicators

Figure 2 shows age-specific changes in height, weight, VCI, and the detection rate of poor vision among primary and secondary school students in Yuzhong District, Chongqing, from 2022 to 2025. All indicators increased with age. For height, no marked sex difference was observed before age 12; after age 12, boys were notably taller than girls, and their height tended to stabilize after age 16. For weight, boys’ weight continued to increase until age 17 and then leveled off, whereas girls’ weight remained generally stable at approximately 57 kg after age 14. For VCI, both boys and girls showed an age-related pattern of initial increase followed by stabilization or slight decline, with boys consistently having higher VCI than girls. The detection rate of poor vision increased gradually with age; in both sexes, it rose rapidly between ages 7 and 11, and the detection rate was consistently higher among girls than among boys.

**Figure 2:**
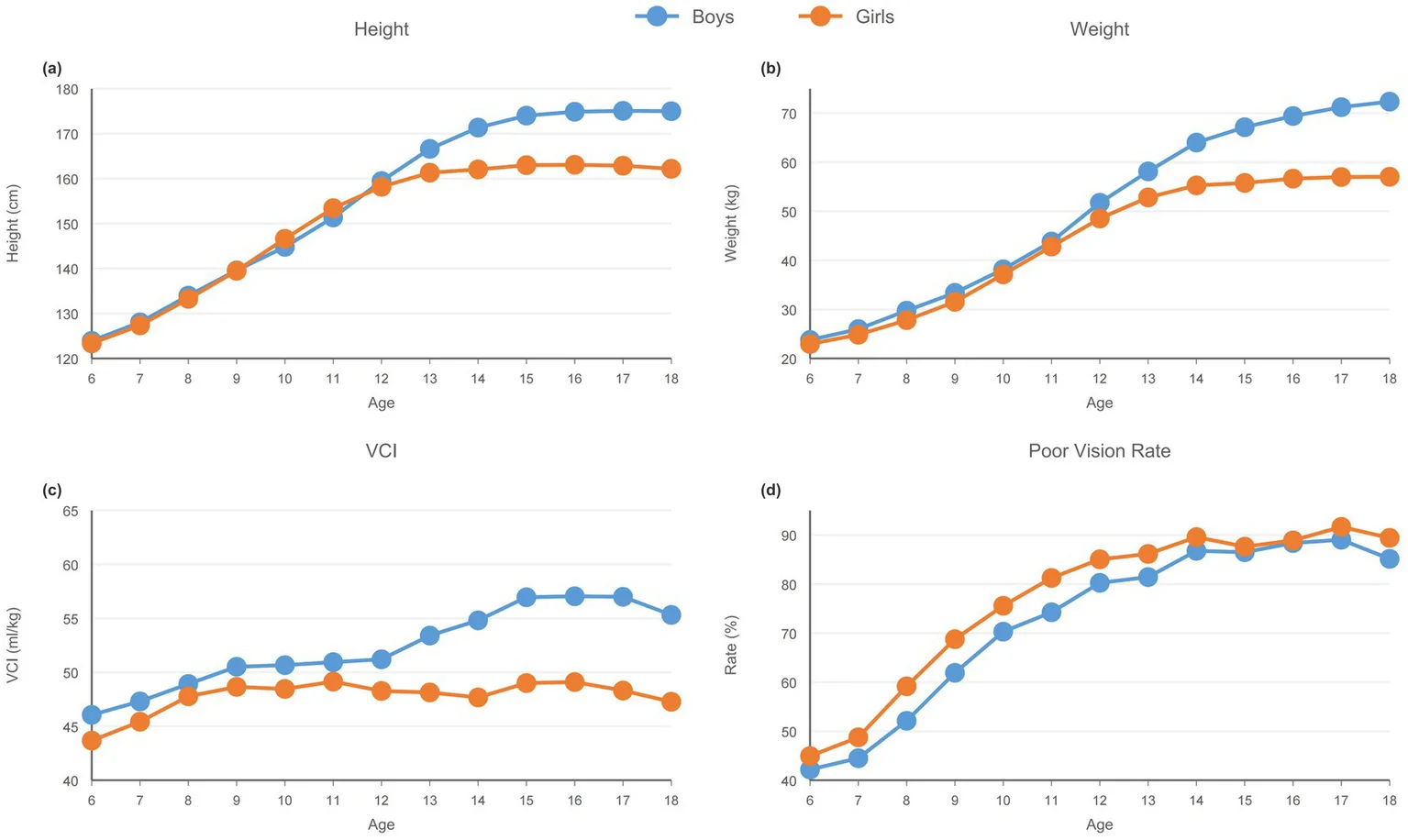
Growth curves of growth and development indicators. **(a)** Height; **(b)** weight; **(c)** VCI; **(d)** poor vision rate.

### 3.3 Characteristics of BMI and VCI trajectory groups

Based on the GBTM results, three distinct trajectories were identified for BMI and four distinct trajectories were identified for VCI in the overall study population. The corresponding model fit indices are presented in Supplementary Table S2. For BMI trajectories, 56.5% of students had relatively low BMI levels with a gradual increase and were classified as the low-level increasing group; 34.0% showed moderate BMI levels with a gradual increase and were classified as the medium-level increasing group; and 9.5% had relatively high BMI levels with a gradual increase and were classified as the high-level increasing group. For VCI trajectories, 23.5% of students had relatively low VCI levels with a gradual increase and were classified as the low-level increasing group; 45.1% had moderate VCI levels with a gradual increase and were classified as the medium-level increasing group; 22.6% had moderate VCI levels with a gradual decrease and were classified as the medium-level decreasing group; and 8.8% had relatively high VCI levels with a gradual decrease and were classified as the high-level decreasing group. Detailed trajectory patterns are shown in Figure 3. To account for potential sex-related differences in BMI and VCI trajectories, sex-stratified sensitivity analyses were additionally conducted. The trajectory patterns identified separately among boys and girls were broadly consistent with those observed in the overall population, supporting the robustness of the primary trajectory classification. Detailed trajectory patterns are presented in Supplementary Figure S1 and Supplementary Table S3.

**Figure 3:**
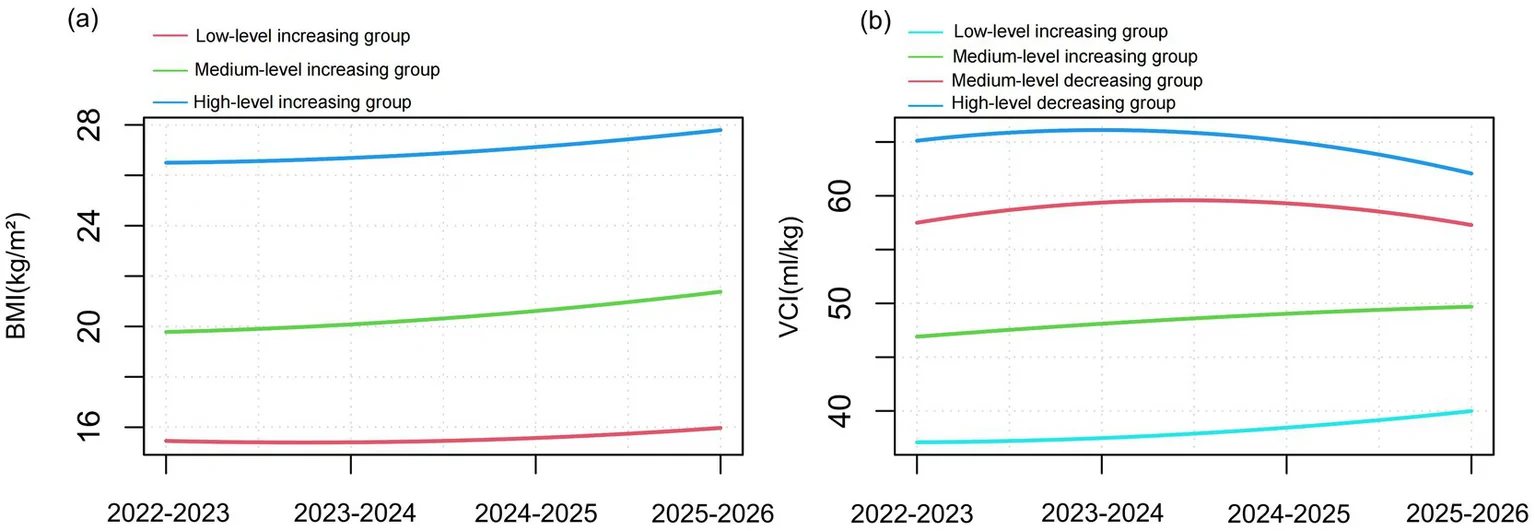
Trajectories of BMI and VCI among primary and secondary school students in Yuzhong District, Chongqing, from the 2022–2023 to 2025–2026 academic years. **(a)** BMI trajectories; **(b)** VCI trajectories.

### 3.4 Detection of poor vision outcomes across different BMI and VCI trajectories

For BMI trajectories, the proportions of students with poor vision in the low-, medium-, and high-level increasing groups were 69.0%, 80.8%, and 86.0%, respectively. For VCI trajectories, the corresponding proportions in the low-level increasing, medium-level increasing, medium-level decreasing, and high-level decreasing groups were 70.2%, 73.7%, 78.7%, and 81.1%, respectively. The differences in the proportions of students with poor vision across BMI and VCI trajectory groups were statistically significant (both *p* < 0.001). Details are shown in Table 2. Comparisons between included and excluded students are presented in Supplementary Table S4.

**Table 2:** Detection rate of poor vision across different trajectory groups.

Level	Overall	Normal vision	Poor vision	*P* value
BMI low-level increasing group	14,017 (56.5)	4,340 (31.0)	9,677(69.0)	<0.001
BMI medium-level increasing group	8,441 (34.0)	1,620 (19.2)	6,821(80.8)	
BMI high-level increasing group	2,348 (9.5)	328 (14.0)	2020 (86.0)	
VCI low-level increasing group	5,817 (23.4)	1736 (29.8)	4,081 (70.2)	<0.001
VCI medium-level increasing group	11,191 (45.1)	2,944 (26.3)	8,247 (73.7)	
VCI medium-level decreasing group	5,603 (22.6)	1,194 (21.3)	4,409 (78.7)	
VCI high-level decreasing group	2,195 (8.8)	414 (18.9)	1781 (81.1)	

### 3.5 Associations of BMI and VCI trajectories and levels with poor vision outcomes

Compared with the low-level increasing group, the medium and high-level increasing BMI trajectory groups had higher odds of poor vision (OR = 1.134, 95% CI: 1.041–1.235; OR = 1.368, 95% CI: 1.165–1.606, respectively). Compared with the reference group, the medium-level decreasing and high-level decreasing VCI trajectory groups had higher odds of poor vision, respectively (OR = 1.117, 95% CI: 1.019–1.226; OR = 1.371, 95% CI: 1.229–1.531), whereas the medium-level increasing trajectory group had lower odds of poor vision, although the association was not statistically significant (OR = 0.968, 95% CI: 0.824–1.138). Details are shown in Table 3. In the nonlinear relationship analysis, BMI level showed no significant nonlinear association with poor vision outcomes (P for nonlinearity = 0.283), whereas VCI level showed a significant dose–response relationship with poor vision outcomes (P for nonlinearity = 0.007). Details are shown in Figure 4.

**Table 3:** Association between different trajectory groups and poor vision.

Variable	OR(95%CI)	*P* value
BMI low-level increasing group	1	
BMI medium-level increasing group	1.134 (1.041–1.235)	0.004
BMI high-level increasing group	1.368 (1.165–1.606)	<0.001
VCI low-level increasing group	1	
VCI medium-level increasing group	0.968 (0.824–1.138)	0.694
VCI medium-level decreasing group	1.117 (1.019–1.226)	0.0188
VCI high-level decreasing group	1.371 (1.229–1.531)	<0.001

Adjusted for sex, age, systolic blood pressure, diastolic blood pressure, and decayed, missing, and filled teeth; school was included as a random intercept.

**Figure 4:**
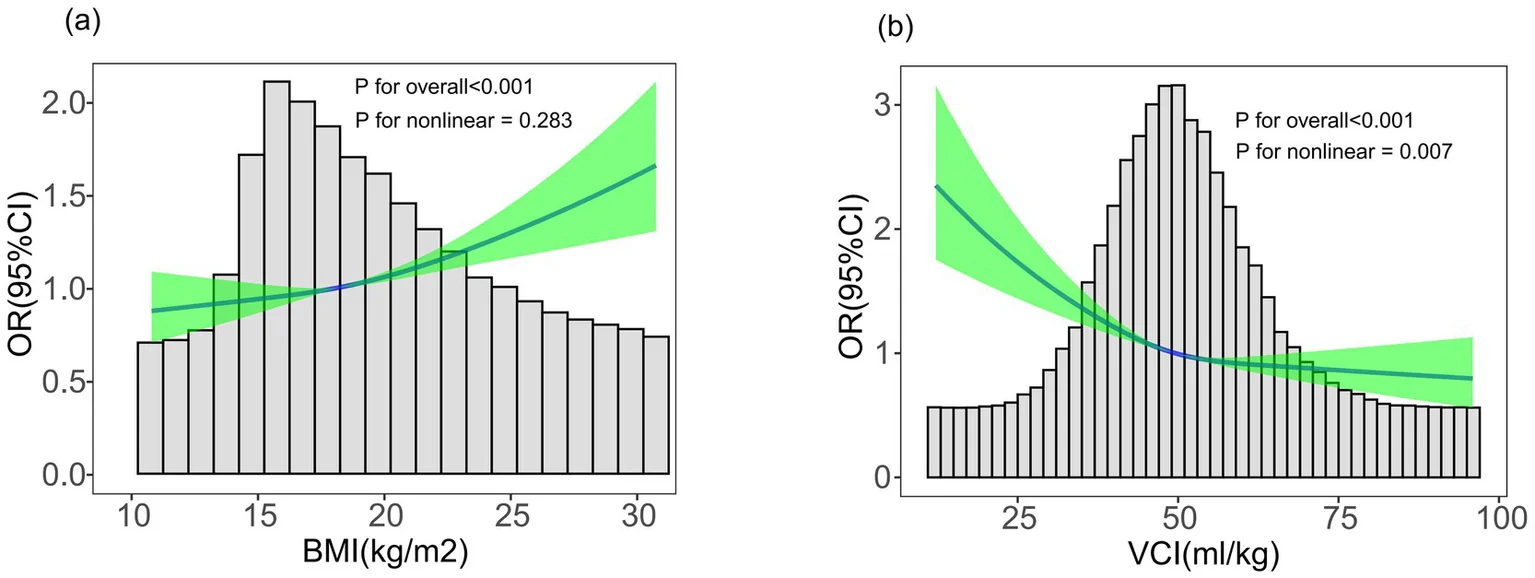
Nonlinear associations between BMI and VCI levels and poor vision. **(a)** Nonlinear association between BMI level and poor vision; **(b)** nonlinear association between VCI level and poor vision.

## 4 Discussion

Based on consecutive health examination data of primary and secondary school students in Chongqing, this study described changes in growth and development indicators among primary and secondary school students in Yuzhong District from the 2022–2023 to 2025–2026 academic years, and identified three developmental trajectories for BMI and VCI, respectively, using GBTM.

Compared with 2022–2023, the overall and age-specific mean height, weight, and vital capacity of boys and girls aged 6–18 years increased during 2022–2026, suggesting continuous improvements in the overall growth and development level and physical function of primary and secondary school students (14, 15). These temporal changes may partly reflect changes in nutritional status, physical activity, and health promotion efforts in recent years, although these factors were not directly measured in the present study. Benefiting from these efforts, the detection rate of overnutrition among primary and secondary school students in Yuzhong District has continued to decline. Height, weight, and VCI all increased with age, with more pronounced growth during puberty. Girls entered puberty relatively earlier, whereas boys showed greater advantages in later adolescence and ultimately reached higher levels, which is consistent with the general patterns of growth and development in children and adolescents (16, 17). The detection rate of poor vision continued to increase with age and was higher in girls than in boys, with a particularly rapid increase between ages 7 and 11 years, which is broadly consistent with the increasing prevalence of poor vision reported among children and adolescents in China (18, 19). Although Yuzhong District has continuously promoted visual health protection among children and adolescents, increasing academic pressure and the widespread use of electronic devices may still adversely affect students’ visual health (20). Therefore, while promoting students’ physical health, vision prevention and control should be further strengthened, with particular attention to primary school students.

Trajectory analysis showed statistically significant differences in the detection rate of poor vision among students with different BMI and VCI trajectories. Multivariable logistic regression indicated that students in the medium and high-level increasing BMI trajectory groups had higher odds of poor vision than those in the low-level increasing group, suggesting that persistently high or rapidly increasing BMI may be associated with higher odds of poor vision. BMI may also serve as a marker of broader lifestyle patterns (21). Previous studies have reported associations between higher BMI and behaviors such as lower physical activity, greater sedentary time, reduced outdoor activity, and greater screen exposure, which may also be related to poor vision (22, 23). However, these behavioral factors were not directly measured in the present study, and therefore their potential contribution to the observed association between BMI trajectories and poor vision cannot be determined. These behavioral factors represent possible, but unverified, explanations for the observed association (24, 25). Compared with single-point measurements, trajectory analysis can better reveal long-term patterns of body weight change and their dynamic associations with visual outcomes.

The difference between the RCS and trajectory analyses may not necessarily indicate a contradiction, but could reflect different aspects of VCI. The RCS analysis may suggest a nonlinear inverse association between current VCI levels and poor vision, whereas trajectory analysis captures longitudinal VCI patterns (26). In our study, the medium-level decreasing and high-level decreasing groups had higher odds of poor vision than the low-level increasing group. This distinction may be particularly relevant in children and adolescents, whose VCI may vary with age, sex, body size, and physical development (27). A higher VCI at a single examination may reflect relatively favorable cardiopulmonary fitness at that time, whereas a persistent decline in VCI may possibly reflect changes in physical development or fitness over time. Therefore, the higher odds observed in the decreasing trajectory groups should not necessarily be interpreted as indicating a harmful effect of higher VCI itself, but may instead reflect an association between longitudinal VCI patterns and poor vision. The inverse association observed in the RCS analysis may be partly explained by the potential relationship between better physical fitness, healthier behaviors, and outdoor exposure (28, 29). Therefore, higher VCI may potentially reflect better cardiopulmonary fitness and healthier behavioral patterns. However, given the observational design, whether VCI itself or related lifestyle factors account for these associations remains uncertain.

Notably, both BMI and VCI trajectories were significantly associated with poor vision, suggesting that poor vision may co-evolve with physical development, physical fitness, and health-related behavioral patterns in children and adolescents (30, 31). Factors such as sedentary learning, excessive near-work activities, insufficient outdoor activity, reduced exercise time, unreasonable dietary patterns, and disrupted sleep rhythms may simultaneously affect BMI, VCI, and visual outcomes (32–34). Therefore, in health promotion efforts for children and adolescents, comprehensive interventions should be implemented from the perspective of coordinated prevention and control of “physical fitness–behavior–vision,” rather than treating vision screening and physical fitness monitoring as separate processes.

This study has several limitations. First, as an observational study, it can only identify statistical associations between variables and cannot establish causal relationships. Second, the poor vision outcome was derived from school-based health examination screening, which cannot fully substitute for clinical refractive examination; moreover, different types of visual impairment, such as myopia, hyperopia, and astigmatism, were not distinguished. Third, potential confounding factors, including eye-use behaviors, outdoor activity, electronic screen exposure, academic burden, and genetic factors, were not fully incorporated, and residual confounding may therefore exist. Fourth, although the trajectory analysis was extended to four academic-year time points by incorporating the complete 2025–2026 data, some students had only three available examinations. In addition, although model selection was supported by multiple fit indices, the relatively limited number of repeated measurements for some students may affect the stability and precision of the estimated trajectories. Therefore, the identified trajectories should be interpreted cautiously as population-level patterns rather than precise individual developmental curves. Furthermore, restricting the trajectory analysis to students with at least three available examinations may have introduced selection bias. Finally, the study population was limited to primary and secondary school students in Yuzhong District, Chongqing, and the generalizability of the findings to other regions and populations requires further validation.

## 5 Conclusion

In summary, growth and development indicators among primary and secondary school students in Yuzhong District, Chongqing, continued to improve from 2022 to 2025, yet the detection rate of poor vision remained high. Both BMI and VCI trajectories were significantly associated with poor vision outcomes, and a significant dose–response relationship was observed between VCI level and poor vision. These findings suggest that, in student health monitoring and myopia prevention and control efforts, comprehensive assessment of physical development, physical fitness, and visual health should be emphasized, and earlier and more targeted health interventions should be implemented for high-risk trajectory groups.

## Data Availability

The dataset contains individual-level health examination information of primary and secondary school students and is subject to privacy protection and ethical restrictions. The data were obtained from the Yuzhong District Primary and Secondary School Health Care Center and cannot be publicly shared to protect the confidentiality of student information. Access to the dataset requires approval from the data provider and compliance with relevant ethical and data protection requirements. Requests to access these datasets should be directed to WR 1395278027@qq.com.
